# Chemopreventive role of *Coriandrum sativum* against gentamicin-induced renal histopathological damage in rats

**DOI:** 10.1515/intox-2015-0015

**Published:** 2015-06

**Authors:** Abhijeet Lakhera, Aditya Ganeshpurkar, Divya Bansal, Nazneen Dubey

**Affiliations:** 1 Drug Discovery Laboratory, Shri Ram Institute of Technology-Pharmacy, Jabalpur, M.P., India; 2 Pharmaceutics Research Laboratory, Shri Ram Institute of Technology-Pharmacy, Jabalpur, M.P., India

**Keywords:** *Coriandrum sativum*, nephroprotection, blood urea nitrogen, creatinine, serum urea

## Abstract

Drug induced nephrotoxicity is one of the most common causes of renal failure. Gentamicin belongs to aminoglycosides, which elicit nephrotoxic potential. Natural antioxidants from plants demonstrate a number of biotherapeutic activities. Coriander is an important medicinal plant known for its hepatoprotective, diuretic, carminative, digestive and antihelminthic potential. This study was designed to investigate whether the extract of *Coriandrum sativum* ameliorates the nephrotoxicity induced by gentamicin in rats. Dried coriander powder was coarsely grinded and subjected to defatting by petroleum ether and further with ethyl acetate. The extract was filtered and subjected to phytochemical and phytoanalytical studies.

Acute toxicity in Wistar rats was determined by the OECD Guideline (423). Animals were divided into four groups. The first group served as positive control, while the second group was toxic control (gentamicin treated). The third and fourth group were treated with the extract (200 and 400 mg/kg gentamicin). After 8 days, the animals were sacrificed and biochemical and histopathological studies were carried out. Phytochemical screening of the extract demonstrated *Coriandrum sativum* to be rich in flavonoids, polyphenolics and alkaloids. Results of acute toxicity suggested the use of 200 mg/kg and 400 mg/kg for *Coriandrum sativum* in the study. *Coriandrum sativum* extract at the dose of 400 mg/kg significantly (*p*<0.01) decreased creatinine levels in the animals, along with a decrease in serum urea and blood urea nitrogen. Treatment with *Coriandrum sativum* extract ameliorated renal histological lesions. It is concluded that *Coriandrum sativum* is a potential source of nephroprotective phytochemical activity, with flavonoids and polyphenols as the major components.

## Introduction

Drug induced nephrotoxicity is one of the most common causes of renal failure. Aminoglycosides are one of the important classes of antimicrobial agents widely used for more than 50 years (Swain & Kaplan-Machlis, 1999). Though novel and more effective antibiotics have been discovered, aminoglycosides are helpful in treating life hostile infections (Mingeot-Leclercq & Tulkens, 1999). Gentamicin is one of the aminoglycosides with nephrotoxic potential. Nephrotoxic effects caused by gentamicin are characterized by augmentation of urea and creatinine levels, along with severe proximal renal tubular necrosis, ultimately leading to renal failure (Al-Majed *et al*., 2002; Cuzzocrea *et al*., 2002). Gentamicin has become a popular substance used to study chronicles about drug-induced renal failure. Gentamicin toxicity is associated with production of reactive oxygen species in the kidney (Reiter *et al*., 2002). Gentamicin interferes also with the phosphorylation process and diminishes levels of ATP in renal tubular cells (Ali *et al*., 2011). This leads to reactive oxygen species induced cell death.

Currently, natural antioxidants from plant source are known to possess a number of biotherapeutic activities. Several plant polyphenols and flavonoids are known to exhibit nephroprotective potential (Liu *et al*., 2012; Gaedeke *et al*., 1996; Adeneye & Benebo, 2008).

Spices, similarly as vegetables, fruits, and medicinal herbs, are known to possess a variety of antioxidant properties (Rice-Evans *et al*., 1997; Madsen & Bertelsen, 1995). *Coriandrum sativum* (Family: Umbelliferae), is an important medicinal plant known for its hepatoprotective, diuretic, carminative, digestive and antihelminthic potential. The plant is also known to treat jaundice (Abderahim *et al*., 2008; Eguale *et al*., 2007; Kiritikar & Basu, 2006).

However, no studies have been done so far to evaluate the antioxidant potential of *Coriandrum sativum* against oxidative stress caused by gentamicin. This study was designed to investigate whether the ethyl acetate extract of *Coriandrum sativum* ameliorates the nephrotoxicity induced by gentamicin in a murine model.

## Materials and methods

### Plant material

*Coriandrum sativum* was obtained from the local market of Jabalpur and authenticated by Dr. A.B. Tiwari, Department of Crop and Herbal Physiology, Jawaharlal Nehru Krishi Vishwavidyalaya, Jabalpur, India. *Coriandrum sativum* was collected in the month of November and dried in shade. It was coarsely powdered and used for preparation of the extract.

### Chemicals and drugs

Gentamicin was purchased from Central Drug House, India. Silymarin was obtained from Microlabs (Pondicherry, India). All the other chemicals were purchased from CDH, India.

### Extraction of the powdered plant material

The plant material (50 g) was coarsely grinded and subjected to defatting by petroleum ether. The defatted material so obtained was extracted separately with ethyl acetate. Finally, the extract was dried at 40°C under pressure and stored at 4°C until subjected to phytochemical screening (Harborne, 1983) and estimation of flavonoids (Slinkard & Singleton, 1977) and polyphenols (Souza *et al*., 2001).

### Animals

Healthy adult male Wistar albino rats between 5 and 6 months of age and weighing about 150–200 g were used for the study. The animals were housed in polypropylene cages and maintained under standard conditions (12 h light: 12 h dark cycle; 25±30 °C; 35–60% humidity). They were fed standard rat pellet diet and water *ad libitum.* All the animal experimental protocols were approved by the Institutional Animal Ethics Committee.

### Acute toxicity study

Acute toxicity studies were performed according to guidelines of the Organization for Economic Cooperation and Development (OECD, 2001). The rats weighing between 150–200 g were in groups of five (n=5). The animals were fasted for 4 h with free access to water only. The Coriandrum sativum extract was administered orally in doses of 2000 mg/kg and 4000 mg/kg to individual groups of rats. The animals were observed over 14 days for mortality and physical/behavioral changes. The dose of 200 mg/kg and of 400 mg/kg of the extract was used in this study. The extract was properly suspended in 1% carboxymethylcellulose and administered to animals via oral route.

### Experimental protocol

Group I served as control group and received distilled water p.o. for eight days. Group II served as gentamicin group. The gentamicin treated group received 100 mg/kg/day gentamicin by intraperitoneal (i.p.) route. Group III and IV received 200 and 400 mg/kg b.w. of ethanolic extract of *Coriandrum sativum.* After dosing on day 8, individual rats were placed in separate metabolic cages for 24h for urine collection to determine urine creatinine content.

### Biochemical determinations

Blood samples were collected via retro-orbital puncture at the end of 24 h, the serum was rapidly separated and processed for determination of serum creatinine, serum urea, blood urea nitrogen (BUN) using Span Diagnostic Kits. Body weight of the animals was also recorded.

### Histopathological studies

The rats were sacrificed and both kidneys were isolated and processed for histopathological examination. The kidneys were excised quickly and fixed in 10% formalin, stained with hematoxylin and eosin and then observed under the microscope for degeneration, fatty changes, necrotic changes and evidence of nephrototoxicity.

### Statistical analysis

The results were expressed as mean ± SEM. Statistical analysis was carried out by using ONE WAY ANOVA followed by *post-hoc* Dunnet's test and *p*<0.01 was considered significant.

## Results

Phytochemical screening of the extract demonstrated *Coriandrum sativum* to be rich in flavonoids, polyphenolics and alkaloids. The yield of the extract was found to be 6.34%. The total amount of phenolic content present in the extract was be 623.32±4.71 mg PE (Pyrocatechol Equivalent)/100 g. By using the standard curve of quercetin (R^2^=0.9998), the total flavonoid content of the extract was found to be 245.76±4.83 mg QE (Quercetin Equivalent)/100 g.

Acute oral toxicity was carried out by the OECD method. *Coriandrum sativum* was found to be safe at the limit dose 4000 mg/kg and 2000 mg/kg, with no mortality in the animals studied. One tenth of this dose, i.e. 200 mg/kg and 400 mg/kg of Coriandrum sativum was used in the subsequent study.

**Figure 1:**
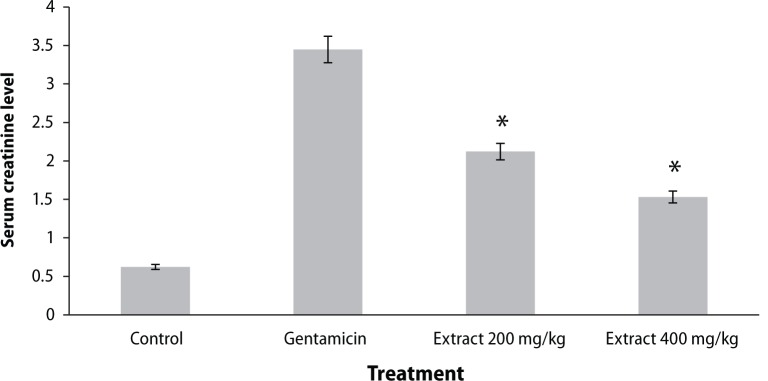
Effect of ethanol extract of *Coriandrum sativum* on serum creatinine in gentamicin treated rats. Results are given as mean ± SEM of five animals in each group. Drug only treated group compared with the extract treated groups. Significance at *p<0.01.

**Figure 2:**
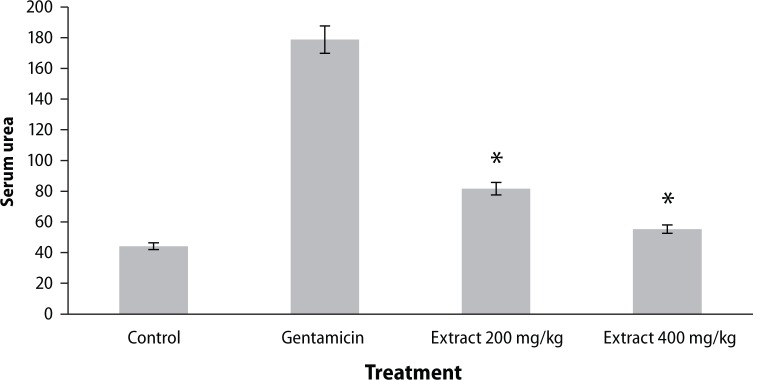
Effect of ethanol extract of *Coriandrum sativum* on serum urea in gentamicin treated rats. Results are given as mean ± SEM of five animals in each group. Drug only treated group compared with the extract treated groups. Significance at *p<0.01.

**Figure 3:**
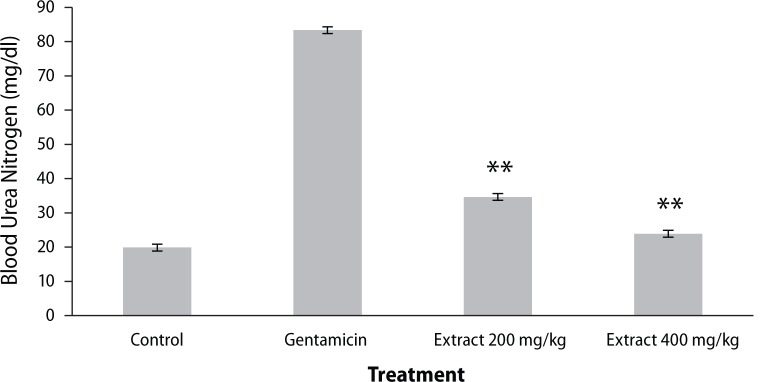
Effect of ethanol extract of *Coriandrum sativum* on blood urea nitrogen in gentamicin treated rats. Results are given as mean ± SEM of five animals in each group. Drug only treated group compared with the extract treated groups. Significance at **p<0.01.

Urine creatinine levels were found to be increased in gentamicin treated animals. *Coriandrum sativum* extract at the dose of 400 mg/kg significantly (*p*<0.01) decreased creatinine levels in the animals (Figure 1). Serum urea and blood urea nitrogen were found to be increased in rats treated with only gentamicin, whereas treatment with the ethanolic extract of the leaf of *Coriandrum sativum* at the dose of 400 mg/kg significantly (*p*<0.01) reversed the effect of gentamicin, indicating nephroprotective activity (Figures 1–3).

**Figure 4:**
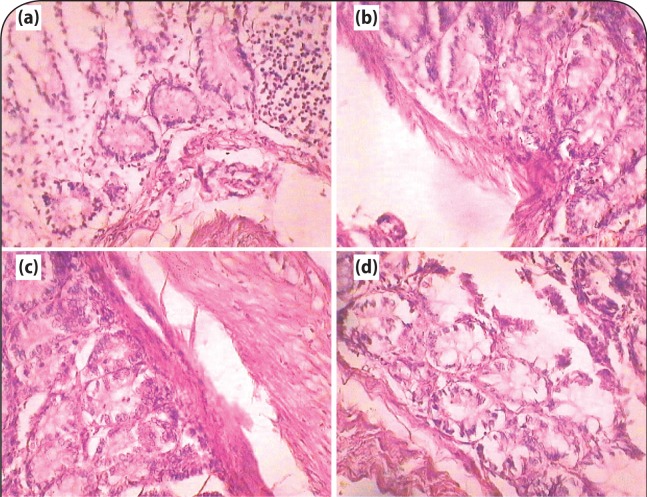
Histopathological evidence of protective effect of *Coriandrum sativum* extract on wistar rats (a) Control (b) Toxicant (c) Extract treated 200 mg/kg, po (d) Extract treated 400 mg/kg, po.

Histopathological studies also revealed the protective effect of B. monosperma extract on the kidney of gentamicin treated rats. Gentamicin induced nephrotoxicity was associated with glomerular and tubular damage, observed by destruction of the tubular lumen. However, treatment with the extract of *Coriandrum sativum* ameliorated renal histological lesions. This effect was more profound in animals treated with 400 mg/kg of ethanol extract of Coriandrum sativum (Figure 4).

## Discussion

Gentamicin, one of the clinically effective antibiotics, is widely used in treatment of gram negative bacterial infections. Scientific studies have shown that gentamicin augmented the production of superoxide, peroxynitrite and peroxyl radicals in renal cortical mitochondria. Furthermore, lipid peroxidation also promotes damage to the structure and function of membranes (Mohammed & Sadeghnia, 2009; Kaloyanideset, 1984; Khan *et al*., 2009; Kuhad *et al*., 2006). Plant-based pharmaceuticals have derived a lot of attention in management of various diseases and disorders.

Natural antioxidants are reputed for their ability to defend cells and the organism from damage attributable to oxidative stress, considered a basis of aging and degenerative diseases. The antioxidants present in food, and especially in vegetables, are phenolic compounds (phenolic acids and flavonoids), carotenoids, tocopherol and ascorbic acid (Cazzi *et al*., 1997; Elmastasa *et al*., 2007). In the present work, the chemopreventive role of Coriandrum sativum against gentamicin-induced renal histopathological damage was studied in the murine model. *Coriandrum sativum,* an important medicinal plant, is known for its hepatoprotective, diuretic, carminative, digestive and antihelminthic potential. Moreover, the plant was also reported to treat jaundice (Abderahim *et al*., 2008; Eguale *et al*., 2007; Kiritikar & Basu, 2006).

The present work was designed to assess the protective effect of *Coriandrum sativum* on gentamicin induced nephrotoxicity in rats. In the present study, gentamicin treatment caused nephrotoxicity, as apparent from the biochemical and histological examinations. Treatment with *Coriandrum sativum* extract prevented a rise of urea, creatinine and blood urea nitrogen in serum. The gentamicin treated rat liver demonstrated the presence of inflammatory anthology and cell necrosis, whereas the treated groups showed no necrosis and the presence of negligible inflammatory surroundings along with normal renal architecture, manifesting the chemoprotective effect against gentamicin induced nephron toxicity. The overall histopathological inspection established the protective effect of the extract on the structural anatomy of the kidney.

It can thus be concluded that *Coriandrum sativum* is a source of phytochemical nephroprotective potential, with flavonoids and polyphenols as the most important constituents. However, more systemic studies are necessary to demonstrate its protective effects in humans.
